# A Randomised, Cross-Over Study to Estimate the Influence of Food on the 25-Hydroxyvitamin D_3_ Serum Level after Vitamin D_3_ Supplementation

**DOI:** 10.3390/nu8050309

**Published:** 2016-05-20

**Authors:** Etienne Cavalier, Bernard Jandrain, Monte Coffiner, Stéphanie Da Silva, Sophie De Niet, Francis Vanderbist, Jean-Claude Souberbielle

**Affiliations:** 1 Department of Clinical Chemistry, University of Liège, CHU Sart-Tilman, Liège 4000, Belgium; 2 Department of Clinical Pharmacology, ATC SA, Liège 4000, Belgium; bernard.jandrain@atc-pharma.be; 3 Clinical Department, Laboratoires SMB SA, Brussels 1080, Belgium; mcoff@smb.be (M.C.); sdsil@smb.be (S.D.S.); sdeni@smb.be (S.D.N.); fvdbi@smb.be (F.V.); 4 Laboratoire d’Explorations Fonctionnelles, Hôpital Necker-Enfants Malades, Paris 75014, France; jean-claude.souberbielle@nck.aphp.fr

**Keywords:** 25-hydroxyvitamin D_3_, absorption, high fat, fasting

## Abstract

Vitamin D_3_ is known to be liposoluble and its release could be a factor limiting the rate of absorption. It was presumed that the presence of fat could favor absorption of vitamin D_3_. However, as bioavailability is related not only to the active molecules but also to the formulations and excipients used, the optimization of the pharmaceutical form of vitamin D_3_ is also important. The objective of this study was to evaluate if there is a food effect on absorption when a high dose of vitamin D_3_ is completely solubilized in an oily solution. In the present cross-over study, 88 subjects were randomized and received a single dose of 50,000 IU of vitamin D_3_ in fasting state or with a standardized high-fat breakfast. Assessment of serum concentrations of 25 hydroxyvitamin D_3_ (25(OH)D_3_) was performed three, five, seven, 14, 30 and 60 days after supplementation. In fed and fast conditions, the 25(OH)D_3_ serum concentrations were significantly higher than the baseline value three days after administration and remained significantly higher during the first month. No significant difference between fasting *vs.* fed conditions was observed. It is therefore concluded that the vitamin D_3_ absorption from an oily solution was not influenced by the presence or absence of a meal.

## 1. Introduction

The importance of vitamin D in health is well documented in terms of its effect on bone, muscle strength, fracture and fall risks. Recently there has been a growing body of evidence that vitamin D may play a role in immune function, cardiovascular health and cancer prevention [1,2,3]. However, it has also been recognized that vitamin D insufficiency remains common in children and adults [4]. The essential question of how much vitamin D is needed for optimal bone and global health remains unresolved. Additionally, the 25-hydroxyvitamin D_3_ (25(OH)D_3_) response to supplementation with vitamin D_3_ varies widely among individuals. Several factors are known to contribute to this variability including genetic variants in the vitamin D binding proteins and in the enzyme that hydroxylates vitamin D in the liver but also the starting serum concentration of 25(OH)D_3_, the body mass index, the age and the serum albumin concentration [5,6]. The presence of a meal and the fat content of that meal may also be important. Because vitamin D is poorly soluble in water, it is hypothesized that absorption would be improved if patients were instructed to take their vitamin D supplement with a meal [7,8,9,10,11]. Concerning vitamin D bioavailability in supplements, the effect of the vehicle substance used was recently reviewed. When the information was combined, oil-soluble vehicles produced the greatest rate of change in mean serum 25(OH)D per 100,000 IU. However, there were discrepancies among the studies which identify important areas for future research [12].

In this new randomized, open, cross-over study, the objective was to determine whether a single dose of 50,000 IU of vitamin D_3_ with food improves the absorption and increases serum concentrations of 25-hydroxyvitamin D_3_.

## 2. Materials and Methods

### 2.1. Methodoloy

This was an interventional, randomized, open, two-treatment, two-period, cross-over study. The study was conducted in one site in Belgium. This trial was reviewed and approved by an Independent Ethics Committee and by the Belgian Competent Authorities (EudraCT No. 2014-003779-48). This study was performed in accordance with the ethical principles that have their origin in the Declaration of Helsinki and that are consistent with Good Clinical Practice (GCPs/ICH E6-Step 5) and the requirements according to the National Drug Law in effect in Belgium in which the study was performed.

**Figure 1:**
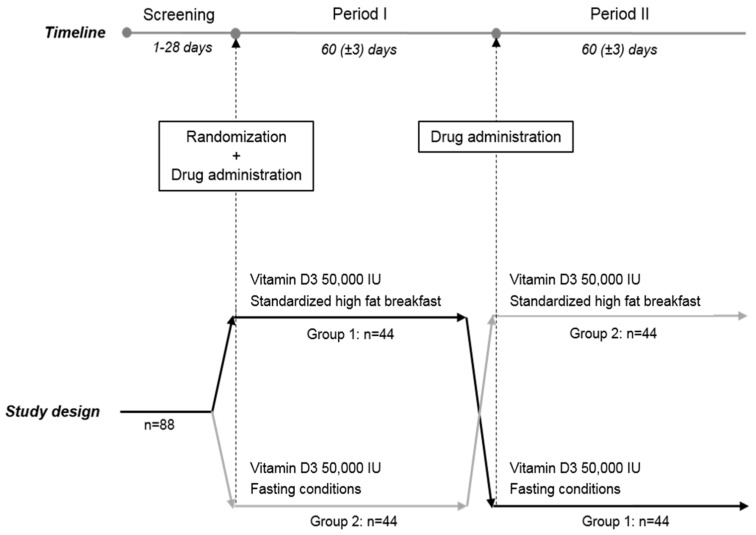
Study design.

The study began in the fourth week of October 2014 and ended in March 2015, a period of the year during which cutaneous synthesis of vitamin D from UV-B sunlight was negligible in Belgium. 88 subjects were included and randomized into two groups of 44 subjects. A screening visit was performed to check the eligibility of each subject in the study. Subjects meeting all the inclusion and none of the exclusion criteria were randomized in one of the two sequences of treatment. The study extended over two periods of 60 ± 3 days. During each period the subject returned to the site for blood sampling on days 3, 5, 7, 14, 30 and 60. The study design is summarized in Figure 1.

Each subject received orally 50,000 IU of vitamin D_3_ solubilized in an oily solution (two ampoules each containing 25,000 IU of D-CURE^®^ provided by Laboratoires SMB SA, Brussels, Belgium) on the first day of each period with or without high-fat breakfast according to the randomization sequence. 

The nutrient composition of the breakfast given is shown in Table 1. Breakfast included two fried or scrambled eggs, two strips turkey, two slices of toast with two pats of butter, 100 g fried potato and one cup of tea or coffee.

Vitamin D_3_ was taken at the study site. The investigator or designee checked that the subjects took the treatment correctly. This was ensured by checking that the ampoules were empty after intake. The subjects stayed at the study site during the 4 h after the vitamin D_3_ administration and returned to the site for the second intake (50,000 IU) 60 ± 3 days after. Both vitamin D_3_ intakes were therefore separated by a wash-out period of 60 days.

### 2.2. Study Population

Caucasian (defined as European and North African), male and female healthy volunteers aged from 18 to 55 years inclusive, with a 25(OH)D_3_ concentration ≥10 ng/mL and ≤20 ng/mL and a BMI between 18 and 25 kg/m^2^ inclusive were selected. They all gave their written, informed consent to participate in this trial.

The main exclusion criteria were any unstable clinically significant immunological, neoplastic, endocrine, hematological, hepatic, cardiac, renal, gastrointestinal, neurological or psychiatric abnormalities or medical disease; past or current granulomatosis, sarcoïdosis, urinary lithiasis, renal insufficiency, osteomalacia; abnormal digestive functions and abnormal thyroid function. Subjects who had a serum creatinine >150 μmol/L and an albumin corrected serum calcium >2.65 mmol/L were excluded at screening. Finally, subjects who used a UV light solarium or any type of vitamin D supplement within two months before the screening visit or planned to travel outside European countries during the study were excluded. Subjects were not allowed to take concomitant medications that were expected to interfere with the interpretation of study data.

### 2.3. Laboratory Assessment

The following tests were performed at the screening visit: RBC, platelets, hematocrit, hemoglobin, WBC and differential count, sodium, potassium, urea, albumin, SGPT/ALAT, SGOT/ASAT, ALP, GGT, total bilirubin, creatinine, calcium, phosphate, TSH and blood β-HCG pregnancy test. A prick finger blood test for a glucose level was performed to confirm the fasting status at each period. All the analyses were performed at the ISO 151198 clinical chemistry laboratory of the University of Liège (Belgium). The LC MS/MS VDSP traceable method was used for serum 25(OH)D measurement as described previously [13]. The samples were measured with the MassChrom^®^ 25-OH-Vitamin D_3_/D_2_ LC MS/MS kit (Chromsystems, Gräfelfing, Germany) including 3-epi-25-OH-Vitamin D_3_ upgrade on the AB SCIEX QTRAP^®^ 5500 system (AB Sciex, Framingham, MA, USA). This method is able to measure the serum 25(OH)D_2_ and 25(OH)D_3_ separately from their epimeric forms 3-epi-25(OH)D. In the following analysis, only the serum 25(OH)D_3_ concentration was considered as the 25(OH)D_2_ concentration was <1 ng/mL in each sample.

### 2.4. Statistical Methods

The statistical tests were one-tailed and the significance threshold retained was 2.5%. The one-sided 97.5% confidence interval of the difference between the means in the two groups was calculated. The statistical analysis was performed using SAS/STAT software version 9.4. (SAS Institute Inc., Cary, NC, USA) of the SAS system for Windows. Baseline value was the serum 25(OH)D_3_ level measured at predose on the first day of each period. The serum concentrations of 25(OH)D_3_ at each time point were compared by a mixed model. Fixed factors were the administration of food, period, sequence and random factors were baseline (day 1 predose), BMI and subject × sequence interaction. Change from baseline to day 3, 5, 7, 14, 30 and 60 of each period was calculated by subtracting the baseline value (day 1 predose) from the value at each time point.

## 3. Results

All randomized subjects (88) completed the two phases of this cross-over study.

The main analysis was conducted on the ITT analysis set. Results were confirmed in two robustness analyses differing by the imputation methods of missing data and in the per protocol subset.

The main demographic characteristics of the subjects are presented in Table 2. As Vitamin D_3_ was taken under the supervision of the study personnel, compliance was 100%.

### Evaluation of 25(OH)D_3_

After a single administration of 50,000 IU/mL of vitamin D_3_ under fasting conditions or with a high-fat meal, the serum concentration of 25(OH)D_3_ rapidly increased after three days (25.1 ± 4.3 *vs.* 25.1 ± 4.9 ng/mL), reached a plateau from day 3 to day 14 (25.6 ± 4.3 *vs.* 25.7 ± 4.6 ng/mL) and decreased until day 60 (Table 3). After 60 days, the 25(OH)D_3_ serum level was close to the baseline value measured before vitamin D_3_ administration (17.2 ± 3.6 *vs.* 17.8 ± 4.0 ng/mL). The increment was similar when the single dose of vitamin D_3_ was taken with a high-fat meal or in fasting conditions. There were no significant group differences in 25(OH)D_3_ concentrations at either three, five, seven, 14 or 30 days (*p* > 0.059) after a single administration of 50,000 IU/mL of vitamin D_3_.

**Figure 2:**
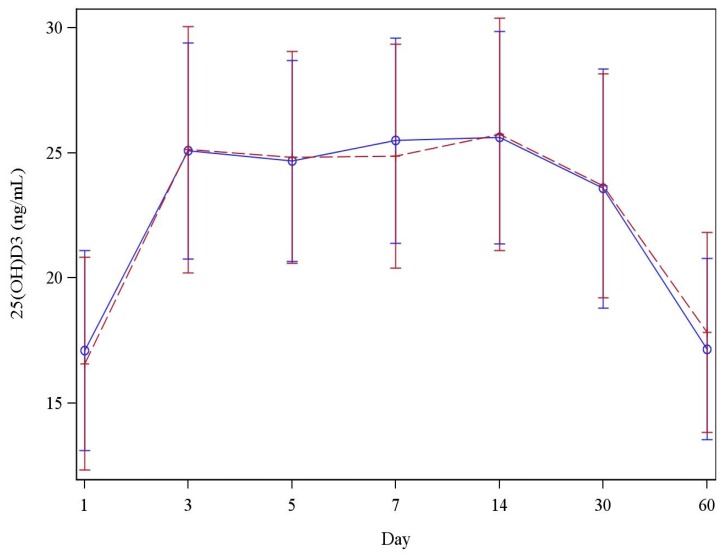
Evaluation of serum 25(OH)D_3_ concentrations following a single dose of vitamin D_3_ supplementation. Solid line: Fasting conditions; Dashed line: High fat conditions.

Similarly, no effect of fasting *versus* fed conditions was observed for the Area Under the Curve calculated after 30 days (AUC_D1–D30_) and 60 days (AUC_D1–D60_) (Figure 2). The estimated difference between means was not significant (*p* > 0.039). AUC_D1–D30_ was 222.2 ± 93.3 ng × 24 h/mL in the fasting period and 236.4 ± 93.6 ng × 24 h/mL in the high-fat period and AUC_D1–D60_ was 319.9 ± 193.1 ng × 24 h/mL in the fasting period and 361.9 ± 187.3 ng × 24 h/mL in the high-fat period.

## 4. Discussion

In the present study, we found no difference between the increase in 25(OH)D_3_ serum concentrations when vitamin D_3_ was taken from an oily supplement in the presence or absence of a meal. The study was well designed; all subjects received their vitamin D_3_ supplement under both conditions (fasting and with a high-fat meal); the meal contained 52.77 g of fat with a high MUFA:PUFA ratio (1.96); the 25(OH)D_3_ level was not significantly different at baseline; and the obtained mean increase in 25(OH)D_3_ and its variability were as observed in other studies performed with the same vitamin D_3_ supplement, which confirms the reliable and reproducible absorption [14,15].

Interestingly, recent studies found different results from ours and suggested that taking a vitamin D supplement with the largest meal of the day improved vitamin D absorption. In a randomized, placebo-controlled study including 64 subjects, Raimundo *et al.* concluded that a single oral dose of a powder capsule of 50,000 IU vitamin D_3_ given with food increased the 25(OH)D_3_ serum concentrations after two weeks [8]. They observed that the mean increase was larger when the meal contained at least 15 g of fat. Another study conducted by Niramitmahapanya *et al.* in 152 healthy men and women concluded that diets rich in monounsaturated fatty acids (MUFA) may improve the effectiveness of powder tablets of vitamin D_3_ supplements while those rich in polyunsaturated fatty acids (PUFA) may decrease the response [10]. A single-dose study conducted by Dawson-Hughes *et al.* in 50 subjects after supplementation with 50,000 IU powder tablets of vitamin D_3_ compared the influence of three different fat-containing meals [11]. The author also concluded that the presence of fat in a meal significantly enhances absorption of vitamin D_3_, but the MUFA:PUFA ratio of the fat in that meal does not influence its absorption.

It is interesting to note that in these studies showing a food effect on vitamin D absorption, the matrix of the supplement was powder contrary to the present study where the vitamin D_3_ was solubilized in an oily solution [7,8,10,11]. A systematic review performed recently by Grossmann *et al.* confirmed that vitamin D in an oil vehicle produced a greater 25(OH)D response than vitamin D in a powder or in an ethanol vehicle [12]. Vitamin D_3_ is known to be liposoluble and its release could be a factor limiting the rate of absorption. It was presumed that the presence of fat could favor absorption of vitamin D_3_. However, as bioavailability is related not only to the active molecules but also to the formulations and excipients used, the optimization of the pharmaceutical form of vitamin D_3_ is also important. In the present study, the hypothesis was that the solubilization of vitamin D_3_ in oil could help to guarantee that patients reach the expected bioavailability independently of the concomitant intake of food.

## 5. Conclusions

In the present study, we can conclude that the bioavailability of a high single dose of vitamin D_3_ (50,000 IU) administered as an oral oily solution was not influenced by the presence or absence of a meal.

## Figures and Tables

**Table 1:** Composition of the breakfast.

Composition	High-Fat Breakfast
Total fat	52.7 g
Saturated fatty acid (SFA)	11.4 g
Polyunsaturated fatty acid (PUFA)	12.35 g
Monosaturated fatty acid (MUFA)	24.25 g
Total carbohydrates	48.72 g
Total protein	28.95 g
Total calories	782 kcal

**Table 2:** Demographic data of the 88 subjects included in the study.

Variables	All Subjects (*n* = 88)
Male, *n* (%)	37 (42)
Female, *n* (%)	51 (58)
Age, year, Mean ± SD	31.3 ± 8.8
min-max; median	19.0–55.0; 28.0
BMI, kg/m^2^, Mean ± SD	22.3 ± 2.0
min-max; median	18.2–25.0; 22.2
25(OH)D_3_, ng/mL, Mean ± SD	16.5 ± 2.3
min-max; median	11.0–20.0; 17.0

**Table 3:** Serum 25(OH)D_3_ concentrations (ng/mL) following a single dose of vitamin D_3_ supplementation.

Blood Sampling Day	Fasting (*n* = 88)		High Fat (*n* = 88)	
	Mean ± SD	Min–Max	Mean ± SD	Min–Max
Baseline (day 1)	17.1 ± 4.0	9.0–26.0	16.6 ± 4.2	6.0–26.0
Day 3	25.1 ± 4.3	18.0–37.0	25.1 ± 4.9	14.0–40.0
Day 5	24.7 ± 4.0	15.0–35.0	24.8 ± 4.2	15.0–35.0
Day 7	25.5 ± 4.1	17.0–39.0	24.9 ± 4.5	16.0–37.0
Day 14	25.6 ± 4.3	18.0–39.0	25.7 ± 4.6	16.0–37.0
Day 30	23.6 ± 4.8	15.0–44.0	23.7 ± 4.5	14.0–35.0
Day 30	17.2 ±3.6	11.0–26.0	17.8 ± 4.0	10.0–30.0
